# Is It Stevens–Johnson Syndrome or MIS-C with Mucocutaneous Involvement?

**DOI:** 10.1155/2021/1812545

**Published:** 2021-12-24

**Authors:** Abdollah Karimi, Elham Pourbakhtyaran, Mazdak Fallahi, Fereshteh karbasian, Shahnaz Armin, Delara Babaie

**Affiliations:** ^1^Pediatric Infections Research Center, Research Institute for Children's Health, ShahidBeheshti University of Medical Sciences, Tehran, Iran; ^2^Department of Allergy and Clinical Immunology, Mofid Children's Hospital, ShahidBeheshti University of Medical Sciences, Tehran, IR, Iran; ^3^Department of Pediatric Gastroenterology and Hepatology, Namazi Hospital, Shiraz University of Medical Sciences, Shiraz, Iran

## Abstract

**Background:**

Severe acute respiratory syndrome coronavirus-2 (SARS-COV-2) can be present in the form of multisystem inflammatory disease in children.

**Case Presentation:**

A 25-month-old boy presented with fever, malaise, diffuse maculopapular rashes, and mucosal involvement during the COVID-19 pandemic. He was first diagnosed with Stevens–Johnson syndrome (SJS). Further evaluation revealed lymphopenia, thrombocytopenia, and elevated levels of C-reactive protein (CRP), ferritin, and fibrinogen. This was followed by a positive polymerase chain reaction (PCR) test for COVID-19. In addition to receiving initial care for SJS, he was treated for MIS-C, which led to his recovery after four days.

**Conclusion:**

COVID-19 infection should be considered in children with fever and dermatological features during the pandemic because it may cause different features of the multisystem inflammatory syndrome in children (MIS-C), suggestive of delayed hyperimmune response.

## 1. Introduction

A novel coronavirus, i.e., severe acute respiratory syndrome coronavirus-2, emerged in December 2019, which led to a pandemic in March 2020 [1]. According to the primary reports, the pediatric population was at a low risk [1, 2]. In April 2020, however, the Pediatric Intensive Care Society of the United Kingdom released an alert regarding an increased number of children tested positive for COVID-19 [1]. Several case reports and small series also emphasized the presentation of an acute illness with multiorgan failure and shock [1, 3, 4]. For instance, Riphagen et al. [5] conducted a research on eight children presented as the first report of hyperinflammatory conditions in the pediatric population due to COVID-19. All eight children presented with similar symptoms such as fever, conjunctivitis, peripheral edema, extremity pain, diarrhea, vomiting, and abdominal pain. They also experienced refractory shock, but none of them experienced significant respiratory involvement. The most recent case reports described pediatric patients presenting with refractory shock symptoms resembling toxic shock syndrome (TSS) rather than Kawasaki disease (KD) [6, 7]. Moreover, patients with COVID-19 and a hyperinflammatory state shared similar cytokine profiles, lymphocyte counts, and levels of inflammatory markers, which made hemophagocytic lymphohistiocytosis (SHLH)/macrophage activation syndrome (MAS) important in differential diagnosis [8, 9].

Although mucocutaneous manifestations are not among the top clinical manifestations of COVID-19 infection in the pediatric population, they are an important clinical manifestation of multisystem inflammatory syndrome in children (MIS-C) [10]. There is a wide range of skin manifestations including Stevens–Johnson syndrome (SJS)/toxic epidermal necrolysis (TEN), which is presented by blistering of the skin and mucous membranes as well as a prodromal phase [10]. The prodromal symptoms are often mistaken for an upper respiratory tract infection. Mucosal involvement affects oral, ocular, genitourinary, and anal sites.

The present study aims to describe a patient with fever, rash, and mucosal involvement admitted with the primary diagnosis of SJS, but finally diagnosed as MIS-C due to organs' involvement and positive polymerase chain reaction (PCR) result for COVID-19.

## 2. Case Report

**Figure 1:**
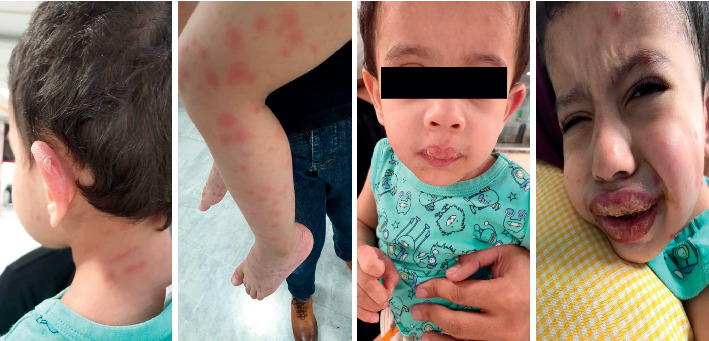
Skin lesions in the patient upon arrival at the hospital.

**Figure 2:**
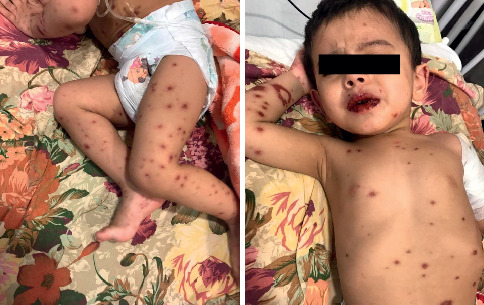
Skin lesions in the patient on the fourth day.

A 25-month-old boy with fever and maculopapular rashes was admitted to Mofid Children's Hospital. His parents explained that fever began three days ago and rashes developed after one day. The rashes started with mild itching in the feet, spreading to the thighs and the genital area on the second day. Despite taking antihistamines, the fever and rashes continued on the third day. The patient presented to the hospital with fever, malaise, poor feeding, mucosal involvement of the mouth, lips, conjunctiva, and maculopapular rashes, which resulted in ulcer and bulla formation (Figure 1). On arrival, he had the following vital signs. Temperature: 39.5°C, blood pressure = 82/10 mmHg, respiratory rate = 28, and pulse rate = 86. The patient's clinical course, blistered skin lesions, and mucosal involvement led to the primary diagnosis of SJS/TEN. Reviewing his medical history revealed that ranitidine was the only medication he had used in the past three weeks. Moreover, his mother had a history of upper respiratory infection (URI) three weeks ago, which was accompanied by low-grade fever and resolved in three days. The patient received supportive care, steroids, and intravenous immune globin (IVIG) based on the primary diagnosis of SJS/TEN. In the meantime, laboratory work up and a COVID-19 PCR test were performed. The results showed white blood cells (WBC) count = 3200/*μ*l (Polymorphonuclear (PMN): 58% and lymph: 41%), hemoglobin (Hgb) = 12.3 gr/dl, and platelet count = 29000/*μ*l. Additionally, the erythrocyte sedimentation rate (ESR) was 36 mm/hr and the CRP level was 58 mg/l. Liver function test, blood urea nitrogen (BUN), creatinine (Cr), albumin, and lactate dehydrogenase (LDH) were within the normal ranges. However, ferritin and fibrinogen levels were elevated (517 *μ*g/L and 615 mg/dL, respectively) and the COVID-19 PCR result was positive. The findings of the chest CT scan were unremarkable. Considering the patient's general conditions, fever, and laboratory findings, MIS-C was diagnosed and atazanavir was added to his treatment. On the following day, his fever subsided and he began to eat and drink. After four days, he was discharged from the hospital with minimal skin lesions and a normal condition (Figure 2). The patient was followed after one week, indicating that his laboratory test results were within the normal ranges and he was doing great.

## 3. Discussion

TEN and SJS are associated with blistering of the skin and mucous membranes, with the incidence of 5.3 (SJS) and 0.4 (TEN) per million children per year in the US population [11]. Prodromal symptoms are often mistaken for an upper respiratory tract infection, and mucosal involvement affects oral, ocular, genitourinary, and anal sites [10].

Primary reports pertaining to SARS-CoV-2 infection in young children indicated that they were spared from severe infection due to a milder disease or lack of detection [12]. In May 2020, Centers for Disease Control and Prevention (CDC) released a health advisory about MIS-C associated withCOVID-19. MIS-C resembles entities such as Kawasaki Disease (KD), Toxic Shock Syndrome (TSS), and secondary hemophagocytic lymphohistiocytosis (SHLH)/macrophage activation syndrome (MAS) [8, 13, 14]. Given the similarities and differences between children with COVID-19-induced hyperinflammation and those with KD, TSS, and HLH, the CDC issued a health advisory establishing standards for the clinical and laboratory definition of MIS-C [7, 8]. Even though mucocutaneous manifestations are relatively uncommon amongst children with COVID-19, they are one of the common clinical manifestations in children with MIS-C, making it crucial for clinicians to reach the MIS-C diagnosis [15].

The present case had diffused maculopapular erythema, conjunctivitis, and dry and red lips involving the oral cavity by the time of admission. Considering the blistering skin lesions and mucosal involvement, SJS-TEN was the main differential diagnosis. However, owing to the COVID-19 pandemic, mother's history of URI in the past three weeks, persistent fever, poor general conditions, leucopenia, lymphopenia, thrombocytopenia, and elevated CRP, COVID-19 PCR testing was requested, which was found to be positive. Further evaluations revealed elevated ferritin and fibrinogen levels. Epidemiological link, patient's age, prolonged fever, laboratory results, two organs' involvement (dermatologic and hematologic), and positive PCR results supported the diagnosis of MIS-C.

Although a previous study provided a report of suspected SJS in an adult patient with confirmed SARS-CoV-2 infection [16], Katlan et al. reported two pediatric cases of COVID-19-associated MIS-C presenting with SJS [17] with impaired liver and kidney functions. Despite all treatments, severe hypoxia continued, leading to one of the case's death. In the current investigation, the patient's kidneys, liver, and pulmonary function were normal. Thus, systemic steroid and IVIG were started as a mainstay of treatment for patients with SJS and MIS-C. Atazanavir was also added to the aforementioned treatment based on the positive PCR result and recommendations of a national consensus for the management of pediatric patients with COVID-19 infection. After four days, a considerable improvement was observed in the patient's conditions and he was discharged after five days

## 4. Conclusion

Despite the initial reports regarding the low incidence of symptomatic COVID infection in children, the higher rate of COVID infection has shown a delayed immune response after COVID-19 infection. As discussed earlier, MIS-C is characterized by mucocutaneous manifestations that are similar to those of many other diseases such as SJS/TEN. However, a wide differential diagnosis should be considered when visiting a child with a mucocutaneous eruption and suspected COVID-19 history. Overall, making the correct diagnosis requires an understanding of the similarities and differences among these conditions.
