# Correction to “Is It Stevens–Johnson Syndrome or MIS-C with Mucocutaneous Involvement?”

**DOI:** 10.1155/crpe/9874027

**Published:** 2025-11-07

A. Karimi, E. Pourbakhtiaran, M. Fallahi, F. karbasian, S. Armin, and D. Babaie, “Is It Stevens–Johnson Syndrome or MIS-C with Mucocutaneous Involvement?,” *Case Reports in Pediatrics*, vol. 2021 (2021), https://doi.org/10.1155/2021/1812545.

In the article titled “Is It Stevens–Johnson Syndrome or MIS-C with Mucocutaneous Involvement?”, there was a spelling error in the last name of the second author, listed as “Elham Pourbakhtiaran” instead of “Elham Pourbakhtyaran.” This is corrected as shown above and in the article.

Furthermore, Figure 1 has been updated to protect the patient's privacy.

We apologise for these errors.

## Figures and Tables

**Figure 1:**
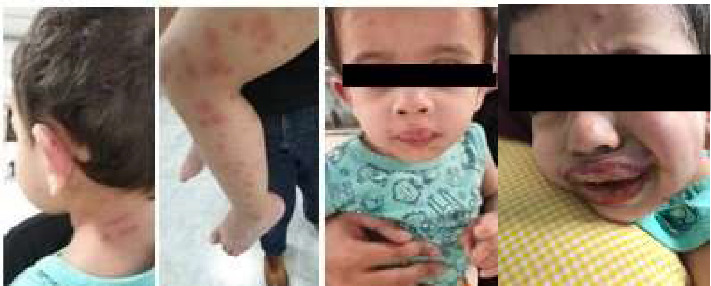
Skin lesions in the patient upon arrival at the hospital.

